# Brain Transcriptomic Dataset During Parturition in Ovoviviparous *Sebastes schlegelii*

**DOI:** 10.3389/fgene.2022.840067

**Published:** 2022-02-04

**Authors:** Likang Lyu, Haishen Wen, Yun Li, Jianshuang Li, Xiaojie Wang, Yijia Yao, Xin Qi

**Affiliations:** Key Laboratory of Mariculture, Ministry of Education, Ocean University of China, Qingdao, China

**Keywords:** transcriptomic data, brain, parturition, ovoviviparous, black rockfish

## Introduction

Parturition is the physiologic process by which a fetus is expelled from the mother to the extrauterine environment. The absolute sequence of events in which neuronal, hormonal, and immune pathways participate seem to be regulated by a serious of factors and integrated cascade mechanisms in both the feto-placental unit and the mother (Ravanos et al., 2015). One of the traditional endocrine views of parturition is the “progesterone withdrawal” theory which states that a fall of progesterone levels and a subsequence estradiol rise lead to the initiation of parturition (Zakar and Hertelendy, 2007). Progesterone is a major factor keeping the uterine environment stable during pregnancy. In mammals including rats, mice, rabbits, and goats, peripheral progesterone levels decrease substantially as parturition approaches (Brown et al., 2004). In rats, progesterone delayed the onset of delivery and reduced the activity of supraoptic and tractus solitarius neurons, which is crucial for parturition initiation (Antonijevic et al., 2000). Another important aspect of parturition is the hypothalamic–pituitary–adrenal (HPA) axis, which was activated during late gestation, and the fetal HPA axis responds to the mother’s HPA axis in cooperation (Ochedalski and Lachowicz, 2004). The neuroplasticity plays an important role in the communication between mother and fetus. In sheep, the downregulation of cell proliferation in the brain during the early postpartum is associated with the establishment of a selective bond with the fetus based on the stabilization of the newborn neurons in the mother (Brus et al., 2010). In rodents, parturition affected both cell proliferation and survival in a different manner across neurogenic zones (Meddle et al., 2007). All the evidence indicated the importance of neuro structure change in parturition.

Parturition is usually used in describing the breeding process of viviparous animals, including mammals. Ovoviviparity, first described in ambiguous terms as another reproductive strategy involving parturition, is different from both oviparity, commonly seen in various species including fish, insects, and birds, and viviparity, which usually happens in most mammals (Lodé, 2012). In oviparous teleost, the neuropeptides including kisspeptin and GnRH were proved to be the trigger of the ovulation at the brain level (Venkatesh et al., 1990). In chub mackerel (*Scomber japonicus*), *kiss1* and *kiss2* show different expression patterns in ovulatory and post-ovulatory periods, as well as *gnrh2* and *gnrh3* (Selvaraj et al., 2012). Similar results were also observed in goldfish (*Carassius auratus*), so a different regulation of two GnRH (cGnRH-II and sGnRH) was tested during ovulation (Canosa et al., 2008). Furthermore, *in vivo* and *in vitro* study showed the stimulatory function of kisspeptins in stinging catfish (*Heteropneustes fossilis*) ovulation (Zhang et al., 2018). On the other hand, steroid hormones synthesized from the gonad, especially 17α, 20β-dihydroxy-4-pregnen-3-one (17, 20β-DHP), were also considered as a key regulator in ovulation. 17, 20β-DHP was able to induced both oocyte mature and ovulation in zebrafish (Klangnurak and Tokumoto, 2017). Nuclear progestin receptor knockout zebrafish could go through final oocyte maturation but suffered from the failure of ovulation (Zhu et al., 2015). Different from the oviparous species, ovoviviparity is fertilization and embryo development occurring in the ovary of the female with facultative lecithotrophic and matrotrophic nutrition at the same time (Balon, 1981; RAP, 2008). The study on ovoviviparous guppy (*Poecilia reticulata*) indicated that different steroid hormones played various roles during the ovary cycle, and 17, 20β-DHP and 17β-estradiol (E_2_) were significantly higher at periparturition (Pertea et al., 2016). Furthermore, it is suggested that the parturition of the guppy triggered by PGF2α was promoted by neuropeptide Arg-vasotocin (Lyu et al., 2021). In the ovoviviparous white-edged rockfish (*Sebastes taczanowskii*), the expression levels of *fshb* and *lhb* were higher during the gestation period, followed by the drastic decrease at parturition (Chen et al., 2018). Kisspeptin, another reproductive neuropeptide regulating the gonadotropin release, varied in the expression level significantly in the ovoviviparous seahorse (*Hippocampus erectus*) during the pregnancy and parturition process (Zhang et al., 2018). However, in teleosts, most research studies were focused on the neuropeptides or the steroid hormones in the brain during ovulation in oviparous species, leading to the lack of knowledge about the ovoviviparous species.

Aiming to identify the potential mechanism of brain signaling in regulating ovoviviparous teleost parturition, the black rockfish (*Sebastes schlegelii*) was employed as a research model in the present study. As an ovoviviparous teleost, black rockfish mate in December, and the oocyte is fertilized in early April the next year. After 25 days of pregnancy, all the fries were delivered at the same time under the control of the reproductive system (Wang et al., 2021; Lyu et al., 2022). Previous research studies have identified the significant expression of prostaglandins endoperoxide H synthase 1/2 (also known as COX-1/2) during black rockfish parturition (Lyu et al., 2022). The synchronized of both embryo development and maternal delivery activation make black rockfish an excellent model to investigate the neuro-regulation mechanism of ovoviviparous teleost parturition. Accordingly, in this study, we provided transcriptomic data from brains of black rockfish at three different time points around parturition. This transcriptomic dataset would be beneficial for the identification of novel genes or pathways involved in the onset of parturition in ovoviviparous teleost. Meanwhile, the dataset can also apply on the molecular mechanism research in different reproductive strategies. Furthermore, bioinformatics software and data analysis tools can be used in this dataset.

## Materials and Methods

### Animal and Sample Collection

Black rockfish (1.4 ± 0.2 kg) were obtained from marine cages located offshore of Dalian in the northern Yellow Sea, Shandong Province, China. All animal experiments in this research were reviewed and approved by the Institutional Animal Care and Use Committee of Ocean University of China prior to the initiation of the study. The studies did not involve endangered or protected species. All experiments were performed in accordance with the relevant guidelines and regulations. Experimental individuals were anesthetized with ethyl 3-aminobenzoate methanesulfonic acid (MS-222, 200 mg/L) and decapitated quickly to minimize animal suffering.

In total, 27 female black rockfish in late pregnancy (late April in 2019) were obtained. Briefly, nine individuals were sampled 2–3 days before parturition (B_I), nine individuals were sampled during parturition (B_II), and nine individuals were sampled 24 h after parturition (B_III). Individuals were terminated as previously described, and brain tissues were sampled and frozen in liquid nitrogen immediately and then stored at −80°C for RNA isolation and further transcriptomic analysis.

### RNA Isolation and Library Construction

Each brain sample including pituitary from 27 individuals was defrosted from -80°C and put into 1 ml Trizol solution (Vazyme, China, Nanjing) with solid-glass beads before homogenization using a V4800 High-throughput tissue lyser (DHSbio, China, Beijing). Then total RNA was extracted according to the Trizol reagent protocol. Qualities and concentrations of total RNA were evaluated using an Agilent 2,100 bioanalyzer system (Agilent Technologies, United States) and NanoDrop (Thermo Fisher Scientific, United States). The RIN (RNA Integrity Number) value of each RNA sample was above 8. To reduce the variety among sample replicates, equal quantities of total brain RNA from three individuals at the same parturition stage were pooled together, resulting in nine RNA pools, B_I_1, B_I_2, B_I_3, B_II_1, B_II_2, B_II_3, B_III_1, B_III_2, and B_III_3. The NEBNext^®^ Ultra™ RNA Library Prep Kit for Illumina^®^ (NEB, United States) was employed to generate nine sequencing libraries according to the manufacturer, and index codes were added to attribute sequences to each sample. The samples were sequenced on an Illumina HiSeq X Ten platform, and 150-bp paired-end reads were generated.

### Transcriptomic Sequence Analysis

The transcriptomic data from nine brain sequencing libraries were used for subsequent analysis, which were obtained by removing the reads of adaptors that were aligned to the reference *Sebastes schlegelii* genome in NCBI (PRJNA516036) with HISAT2, a highly efficient spliced alignment program for mapping RNA-seq reads (Kim et al., 2015). Assemble and quantification analysis was accomplished using the Stringtie package (Pertea et al., 2016). The multiple mapped reads were removed, and the count numbers of unique mapped reads and FPKM (fragments per kilobase per million) were retrieved and normalized with previous references (Li and Dewey, 2011; Anders et al., 2015). Principal component analysis (PCA) was performed *via* the ggplot2 package.

### Differential Analysis and Annotation

Based on the edgeR package, statistical analysis of transcripts was conducted with a cut-off “*p value*” < 0.05 (Robinson et al., 2010). Transcripts with absolute fold change values greater than one were marked as significantly differentially expressed genes (DEGs). DEG annotation was based on the reference *Sebastes schlegelii* genome. The detail list of DEGs in B_II vs. B_I group, B_II vs. B_III group, and B_III vs. B_I group are represented in Supplementary Table S3–5 for further functional annotation.

DEGs were then assigned to Gene Ontology (GO) classification with the aid of the Blast2GO program (Gotz et al., 2008). These gene terms were then enriched on the three GO categories (biological process, cellular component, and molecular function at level 2) using the GOseq R package (Young et al., 2010). Kyoto Encyclopedia of Genes and Genomes (KEGG) pathway enrichment analysis (a database of biological systems, http://www.genome.jp/kegg/) was performed on significant pathway enrichment analysis. We used the clusterProfiler R package to test the statistical enrichment of differential expression genes in KEGG pathways (Kanehisa et al., 2017).

## Preliminary Analysis Report

Transcriptome sequencing of nine sequencing libraries from 27 black rockfish brain tissues was performed. In total, 520,499,566 raw reads and 501,742,988 clean reads were obtained from nine libraries with an error rate under 0.03%. For the majority of each sample, over 90% of the reads were uniquely mapped on the *S. schlegelii* genome and about 3% reads were multiple mapped (Supplementary Table S1). The raw sequences have been deposited in the Short Read Archive (SRA) of the National Center for Biotechnology Information (NCBI) under the BioProject accession number of PRJNA787732.

**FIGURE 1:**
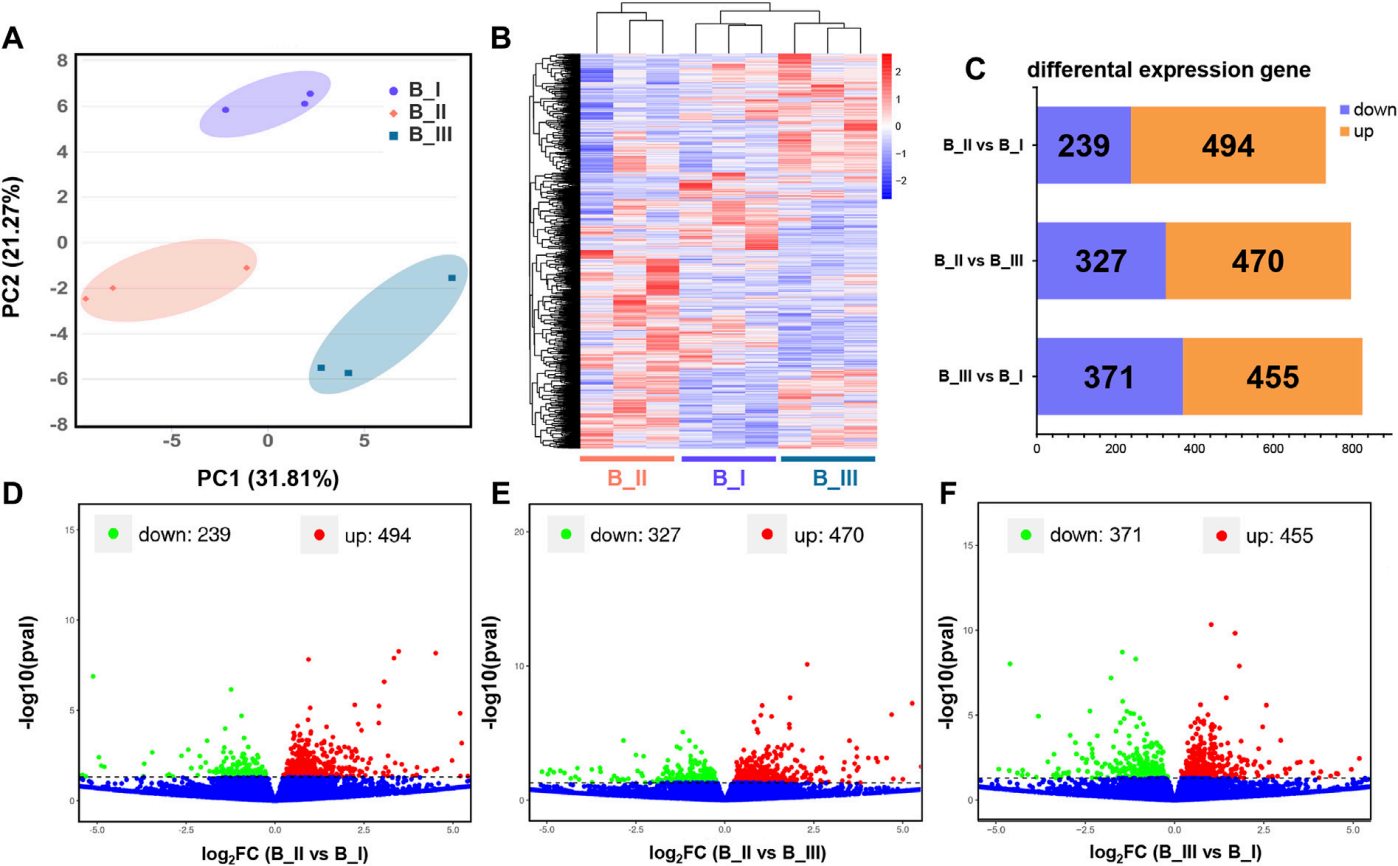
Transcriptional profiles in the brain of black rockfish parturition. Principal component analysis (PCA) **(A)** and heatmap **(B)** of different expression gene (DEG) function clustering of black rockfish brain before parturition (B_I), during parturition (B_II), and 24 h after parturition (B_III). Bar plot **(C)** and volcano plots represented the DEGs in B_II vs. B_I group **(D)**, B_II vs. B_III group **(E)**, and B_III vs. B_I group **(F)**, respectively.

Assemble and quantification analysis was accomplished using the Stringtie package. After that, expression level of these 28,594 transcripts was quantified as FPKM. As shown in Supplementary Table S2, the FPKM of each gene in the present transcriptomic project, as well as the gene locus in the whole chromosome, was presented for further differential expression analysis. Meanwhile, the separated PCA assay was generated by principal component 1 and 2 (PC1 = 31.81% and PC2 = 21.27%) to testify the sample variation. It is suggested that the brain samples from the same parturition stage were clustered closely, while each group was distant (Figure 1A).

Based on previous data, transcript screening was achieved using the edgeR package with a *p* value < 0.05. Totally, 1,835 DEGs were filtrated, including the following: 1) 733 DEGs were obtained in B_II vs. B_I group with 239 downregulated genes and 494 upregulated genes; 2) 797 DEGs were obtained in B_II vs. B_III group with 470 downregulated genes and 327 upregulated genes; and 3) 826 DEGs were obtained in B_III vs. B_I group with 371 downregulated genes and 455 upregulated genes (Figure 1C). The DEG details are listed in Supplementary Table S3. Visualization of *p* value and foldchange distribution of DEGs in each comparison group was presented by volcano plots in Figures 1D–F. A heatmap revealed the transcriptomic pattern of these 1,835 DEGs in three parturition periods (Figure 1B). It is suggested that B_III and B_I had similar expression patterns, which are disparate from B_II.

**FIGURE 2:**
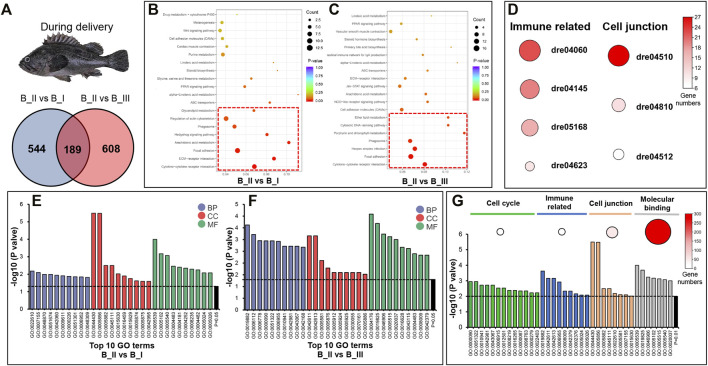
GO and KEGG annotation of DEGs in B_II vs. B_I group and B_II vs. B_III groups. Venn diagram of DEGs in B_II vs. B_I and B_II vs. B_III **(A)**. Bubble chart of KEGG pathways in B_II vs. B_I **(B)** group and B_II vs. B_III group **(C)**; red boxes indicated the significant enriched pathways (*p* value < 0.05). Heatmap of immune-related and cell junction pathways in both B_II vs. B_I group and B_II vs. B_III group; the size of each circus indicated enriched gene numbers in each pathway **(D)**. Top 10 GO terms in three level one categories (biological process, BP; cellular component, CC; and molecular function, MF) of B_II vs. B_I group **(E)** and B_II vs. B_III group **(F)**. GO terms related to cell cycle, immune related, cell junction, and molecular binding in B_II vs. B_I group and B_II vs. B_III group; the size of each circus indicated enriched gene numbers in each GO term **(G)**.

Due to the expression pattern mentioned before, we further presented the DEG annotation result in B_II vs. B_I group and B_II vs. B_III group. As can be seen from the Venn graph, 1,341 DEGs in the sum included 544 DEGs uniquely differentially expressed in B_II vs. B_I group, 608 DEGs in B_II vs. B_III group, and 189 DEGs differentially expressed in both groups (Figure 2A). In an annotation assay *via* the KEGG database, eight significant KEGG pathways (*p* value < 0.05) were enriched in B_II vs. B_I group including *Cytokine-cytokine receptor interaction* (dre04060), *ECM-receptor interaction* (dre04512), *Focal adhesion* (dre04510), *Arachidonic acid metabolism* (dre00590), *Hedgehog signaling pathway* (dre04340), *Phagosome* (dre04145), *Regulation of actin cytoskeleton* (dre04810), *Glycerolipid metabolism* (dre00561) (red box in Figure 2B). Likewise, seven significant KEGG pathways were showed in B_II vs B_III group including *Cytokine-cytokine receptor interaction* (dre04060), *Focal adhesion* (dre04510), *Herpes simplex infection* (dre05168), *Phagosome* (dre04145), *Porphyrin and chlorophyll metabolism* (dre00860), *Cytosolic DNA-sensing pathway* (dre04623), *Ether lipid metabolism* (dre00565) (red box in Figure 2C). The detailed list of these KEGG pathways is shown in Supplementary Table S4. Further analysis showed that four immune-related pathways (dre04060, dre04145, dre05168, and dre04623) were co-enriched significantly in both groups, as well as cell junction–related pathways (dre04510, dre04810, and dre04512). Figure 2D showed the heat plot of DEG numbers of these immune and cell junction related pathways.

GO enrichment results of B_II vs. B_I and B_II vs. B_III are visualized as bar plots in Figures 2F,G, which are the top 10 GO terms (*p* value < 0.05) from the biological process (BP), cellular component (CC), and molecular function (MF) categories. The detailed list of GO terms is presented in Supplementary Table S5. Narrowing down the threshold as *p* value < 0.01, several GO terms were clustered into four functional classifications including the following: 1) cell cycle (*mitotic anaphase* GO:0000090, *anaphase* GO:0051322, *regulation of cell death* GO:0010941, *regulation of apoptotic process* GO:0042981, *regulation of programmed cell death* GO:0043067, *apoptotic process* GO:0006915, *programmed cell death* GO:0012501, *cell death* GO:0008219, *death* GO:0016265, *mitotic M phase* GO:0000087, *mitotic cell cycle phase* GO:0098763, *M phase* GO:0000279, *cell cycle phase* GO:0022403) with 19 DEGs, 2) immune related (*antigen processing and presentation* GO:0019882, *MHC protein complex* GO:0042611, *MHC class II protein complex* GO:0042613, *immune response* GO:0006955, *chemokine activity* GO:0008009, *chemokine receptor binding* GO:0042379, *immune system process* GO:0002376, *transforming growth factor beta-activated receptor activity* GO:0005024, *transforming growth factor beta receptor activity, type II* GO:0005026) with 19 DEGs, 3) cell junction (*cytoskeletal part* GO:0044430, *cytoskeleton* GO:0005856, *intermediate filament* GO:0005882, *intermediate filament cytoskeleton* GO:0045111, *biological adhesion* GO:0022610, *collagen trimer* GO:0005581, *cell adhesion* GO:0007155, *microtubule cytoskeleton* GO:0015630) with 54 DEGs, and 4) molecular binding (*glycosaminoglycan binding* GO:0005539, *oxygen binding* GO:0019825, *tetrapyrrole binding* GO:0046906, *receptor binding* GO:0005102, *protein bindin*g GO:0005515, *hyaluronic acid binding* GO:0005540, *heme binding* GO:0020037) with 272 DEGs. Visualized bar plot of selective functional GO terms is shown in Figure 2H.

## Data Availability

The datasets presented in this study can be found in online repositories. The names of the repository/repositories and accession number(s) can be found in the article/Supplementary Material.

## Supplementary Material

Supplementary Table S1 Overview of transcriptomic data of black rockfish brain.

Supplementary Table S2 Quantification result transcriptomic data of black rockfish brain. Result showed as FPKM for each sample.

Supplementary Table S3 DEG list of B_II vs B_I (*p* value < 0.05), B_II vs B_III (*p* value < 0.05), and B_III vs B_I (*p* value < 0.05).

Supplementary Table S4 Enriched KEGG pathways in B_II vs B_I group and B_II vs B_III group.

Supplementary Table S5 Enriched GO term list of B_II vs B_I group and B_II vs B_III group.
